# HERV Modulation in Colorectal Carcinoma Patients: A Snapshot of Endogenous Retroviral Transcriptome

**DOI:** 10.1002/jmv.70249

**Published:** 2025-02-24

**Authors:** Nicole Grandi, Ching‐Hsuan Liu, Saili Chabukswar, Daniele Carta, Yun Yen, Liang‐Tzung Lin, Enzo Tramontano

**Affiliations:** ^1^ Laboratory of Molecular Virology, Department of Life and Environmental Sciences University of Cagliari Cagliari Italy; ^2^ Department of Microbiology and Immunology, School of Medicine, College of Medicine Taipei Medical University Taipei Taiwan; ^3^ Department of Microbiology & Immunology Dalhousie University Halifax Canada; ^4^ International Ph.D. Program in Medicine, College of Medicine Taipei Medical University Taipei Taiwan; ^5^ Graduate Institute of Cancer Biology and Drug Discovery, College of Medical Science and Technology Taipei Medical University Taipei Taiwan; ^6^ Center for Cancer Translational Research Tzu Chi University Hualien City Taiwan; ^7^ Graduate Institute of Medical Sciences, College of Medicine Taipei Medical University Taipei Taiwan

**Keywords:** colorectal cancer, HERV, human endogenous retroviruses, RNAseq, transcriptome

## Abstract

Human endogenous retroviruses (HERVs) are proviral relics of infections that affected primates' germ line. Many HERV elements retain a residual capacity to encode transcripts and proteins that have been occasionally domesticated for the host physiology. In addition, HERV transcriptional modulation is of great interest to clarify the etiology of complex disorders such as cancer, even if a few studies assessed the specific HERV loci modulated in tumor tissues. In the present work, we used a transcriptomic approach to investigate the specific expression of ~3300 HERV loci in paired tumor and normal tissues of 7 colorectal cancer (CRC) patients. A total of 102 HERVs were significantly modulated in CRC, with a general tendency towards downregulation. Of note, among the 42 upregulated HERVs 23 belonged to the HERV‐H group, that is the most investigated in CRC. De novo transcriptome reconstruction and qPCR validation allowed to identify a transcript from a HERV‐H locus on chromosome Xp22.3 with high specific expression in CRC samples, potentially encoding for a partial Pol protein. These results provide a detailed description of HERV transcriptional variations in CRC and its interindividual variability, identifying a HERV‐H transcript that deserves further investigation for its possible impact on tumor progression.

## 1 Introduction

Colorectal cancer (CRC) is the third most diagnosed cancer as well as the second‐highest cause of cancer‐related deaths globally, with a 15% of 5‐year survival rate for the metastatic form. The etiology of CRC is still unclear, and great attention is dedicated to the possible impact of microbial agents on its onset and progression. These include, on the one hand, the gut microbiota, encompassing about 38 trillion bacteria whose alteration has been observed in CRC [1] and, on the other hand, human endogenous retroviral elements, namely HERVs, whose possible role has attracted a growing attention.

HERVs have been inherited by the human genome from our primate ancestors, which in turn acquired these integrations along millions of years through the infection of their germ line cells by numerous and now extinct retroviruses [2]. These elements constitute approximately 8% of human DNA, and selected members have been domesticated along primate evolution to provide essential physiological roles, being involved (among the others) in pregnancy, innate immunity networking, and brain plasticity [3, 4, 5]. As an example, a member of the HERV‐W group located in locus 7q21.2 encodes for an envelope (Env) protein called Syncytin‐1, which has been coopted to provide its fusogenic activity in the morphogenesis of the placental syncytiotrophoblast, i.e. the site for trophic exchanges between mother and fetus [3]. Given their residual expression and coding potential, HERVs are also highly investigated in several human diseases, including complex disorders such as cancer and autoimmunity. Particularly, HERVs have the potential to trigger tumorigenesis in multiple ways, including the activation of oncogenic pathways through their regulatory elements or the production of retroviral proteins with transforming activity. The latter have been specifically suggested for retroviral Env–that can stimulate tumor cell fusion and invasion through their syncytial activity–and for two Env splicing variants found in some Class II HERV groups and proposed as HERV‐derived oncogenes [3]. Besides having direct transforming activity, the transcriptional de‐repression of HERVs in cancer tissues was shown to trigger innate immunity by the formation of double stranded RNA (dsRNA), through the complementary association of highly similar antisense transcripts, providing immunogenic sequences potentially to be exploited in shock and kill immune‐oncolytic approaches [6, 7, 8].

A number of studies analyzed HERV expression in CRC, focusing especially on the Class I gammaretrovirus‐like HERV‐H group–that is one of the most numerous, counting more than 1000 integrations in the human genome [2, 9]–and on the Class II betaretrovirus‐like HERV‐K (HML2) group, that is, the most recently integrated in humans, with latest acquisitions occurred around 0.2 million of years ago [10, 11]. HERV‐H elements expression has been repeatedly reported in CRC tissues, and the de‐repression of their transcription was shown to influence transcriptional networks relevant to tumor progression [12, 13]. Finally, a recent study reported a correlation between the elevated expression of HERVs and the low responsivity to chemotherapy in a cohort of patients with stage II and III CRC, with a potential prognostic relevance [14].

In the present study, we wanted to enlarge and better detail such studies and investigated the specific expression of around 3300 HERV loci in transcriptomic profiles generated from paired tumor and adjacent normal tissues of seven CRC patients, which were sequenced in triplicate for each condition. The analysis highlighted a certain interindividual variability among CRC patients, and defined a set of HERV loci that were commonly modulated in all CRC samples as compared to healthy controls. Among these, a HERV‐H locus in chromosome X showed high expression levels in the tumor, with the production of some transcripts that were inferred exclusively in CRC tissues.

## 2 Materials and Methods

### 2.1 Clinical Sample and RNA Extraction

Colon cancer specimens and their matched adjacent normal tissues were collected from Taipei Medical University Hospital with approval from the Joint Institutional Review Board of Taipei Medical University. The specimens were preserved in RNA*later* Stabilization Solution (Invitrogen) and homogenized using TissueRuptor (Qiagen). Total RNA was extracted from the tissue samples using TRIzol Reagent (Invitrogen) and treated with DNase I (NEB) according to the manufacturers' instructions, followed by purification using the Monarch Spin RNA Cleanup Kit (NEB). The purified RNA samples were quantified and frozen at −80°C.

### 2.2 RNA Sequencing

Total RNA sequencing with ribosomal RNA removal was performed using the Illumina HiSeq. 3000/4000 System with TruSeq RNA Library Preparation kit v2, HiSeq. 3000/4000 SBS kit, and HiSeq. 3000/4000 PE Cluster kit (Illumina). Conditions are set according to optimized settings for the sequencing of HERV expression, using paired‐ends 150 bp reads length and 35 million reads/sample. The sequencing service was provided by Fulgent Genetics (El Monte, CA, USA).

### 2.3 Processing of Raw RNA Sequencing Data

For each RNA‐seq sample, the corresponding paired fastq files have firstly been subjected to quality control with the software FastQC [15] and then aligned to the reference human genome sequence (GRCh38/hg38 assembly) using STAR aligner, version ﻿2.5.2 [16]. Unmapped reads or reads mapping to multiple genomic position were excluded, and only univocally‐mapped reads were used for subsequent analyses. Python library htseq‐count [17] has been used to quantify the reads mapping to each individual HERV locus included in our HERV data set (HERVdb, ~3300 HERVs) as well as to each human gene reported in Gencode data set, version 34 [18], relying on their univocal genomic coordinates [19, 20, 21, 22, 23, 24, 25, 26]. More in detail, the HERVdb was firstly originated by the comprehensive identification and classification of the most integer HERV integrations in the human genome as performed with RetroTector software [2] and further implemented after the genomic characterization of individual HERV groups, by refining the coordinates of those loci that were already present as well and adding the ones not previously included [20, 22, 26, 27, 28]. The obtained raw counts have been normalized by regularized‐logarithm transformation considering each HERV/gene length and each sample sequencing depth, and used for the generation of unsupervised clustering plots and differential expression analysis with RStudio software, version 1.4.1106 [29]. The same raw counts have been also used to calculate the relative abundance of reads as transcripts per million Kb (TPM) expression values, which normalize the number of reads based on the length of the corresponding HERV/gene.

### 2.4 Clustering and Differential Expression Analyses

Unsupervised clustering and differential expression analyses have been performed on RStudio software [29]. Clustering plots have been generated using the following packages: gplots (version 3.1.3), ggplot2 (version 3.4.2), RColorBrewer (version 1.1‐3), and pheatmap (version 1.0.12).

Differential expression analyses have been conducted with DEseq. 2 package (version 1.38.3) [30] setting as statistical threshold a Benjamini‐Hochberg adjusted *p*‐value (*p*‐adj) ≤ 0.01 and an absolute log2‐fold change (log2FC) ≥ 1. Differentially expressed HERVs and genes have been further classified into upregulated (DEL+) or downregulated (DEL‐) according to their positive or negative fold change as compared to normal tissue, respectively. Results have been represented in a volcano plot using ggplot2 package (version 3.4.2). The eventual colocalization of modulated HERVs with cellular genes (i.e., presence of the HERV as an integration within the gene sequence) has been assessed by the intersection of the former genomic coordinates with the ones of Gencode data set, version 34, through the Data Integrator tool of USCS Genome Browser. Colocalized HERV and genes have been visualized on the latter to evaluate their respective orientation and the exonic/intronic integration of the HERV element.

### 2.5 Transcriptome Reconstruction

Raw RNA‐seq data of tumor and normal samples have been used for the de novo reconstruction of the respective transcriptomes using Trinity toolkit [31]. In particular, merged raw read files from all the CRC samples and all the paired nontumor tissues were considered to generate the transcriptomes representing CRC and healthy conditions, respectively. Reconstructed transcripts have been mapped back to the human genome reference sequence with GMAP mRNA aligner [32] and visualized in the context of the human genome with the Integrative Genomics Viewer (IGV) software [33].

### 2.6 Quantitative Real‐Time PCR

qRT‐PCR was performed in five pairs of the sequenced CRC specimens using the following primers specifically designed to amplify the transcript identified for HERVH‐5290: forward 5′‐TCCTCAATACCTCCCTCTACTACC‐3′, reverse 5′‐GGGATGAAGGGTGCAAAGGA‐3′. Statistical significance (**p ≤ 0.01) was determined by Mann–Whitney test using GraphPad Prism 9.

## 3 Results

### 3.1 RNA‐Seq Profiles From Colorectal Cancer Patients

The present study involved 7 pairs of CRC tumor tissue and an adjacent normal tissue specimens that were sequenced in triplicate, obtaining a total of 42 transcriptomic profiles including 21 tumor samples and 21 matched healthy control samples (Table 1). The individual raw reads' files were subjected to quality control, satisfying the different parameter analyzed (Figure S1).

**Table 1:** Transcriptomic profiles obtained from seven CRC patients in the present study.

Patient^1^	CRC site	TNM^2^	pStage^2^	Differentiation	Sample ID	Condition	RNA‐seq ID
12 (M, 82)	Proximal sigmoid	T2N0M0	1	Well differentiated	FT‐SA51523	Tumor	12T_s1
							12T_s2
							12T_s3
					FT‐SA51524	Normal	12N_s1
							12N_s2
							12N_s3
13 (F, 74)	Sigmoid	TisN0M0	0	Well differentiated	FT‐SA51525	Tumor	13T_s1
							13T_s2
							13T_s3
					FT‐SA51526	Normal	13N_s1
							13N_s2
							13N_s3
14 (F, 69)	Rectal	T2N1M0	3a	Well differentiated	FT‐SA51527	Tumor	14T_s1
							14T_s2
							14T_s3
					FT‐SA51528	Normal	14N_s1
							14N_s2
							14N_s3
15 (F, 61)	Colon (ascending)	T4bN1bM0	3c	Moderately differentiated	FT‐SA51529	Tumor	15T_s1
							15T_s2
							15T_s3
					FT‐SA51530	Normal	15N_s1
							15N_s2
							15N_s3
17 (F, 90)	Colon (ascending)	T4aN2bM0	3c	Moderately differentiated	FT‐SA51531	Tumor	17T_s1
							17T_s2
							17T_s3
					FT‐SA51532	Normal	17N_s1
							17N_s2
							17N_s3
18 (F, 78)	Colon (transverse)	T3N1cM1c	4	Well differentiated	FT‐SA51533	Tumor	18T_s1
							18T_s2
							18T_s3
					FT‐SA51534	Normal	18N_s1
							18N_s2
							18N_s3
20 (F, 28)	Cecum	T2N1aM0	3a	Well differentiated	FT‐SA51537	Tumor	20T_s1
							20T_s2
							20T_s3
					FT‐SA51538	Normal	20N_s1
							20N_s2
							20N_s3

^1^ Gender and age at the time of biopsy are also indicated.

^2^ The TNM classification and pathological stage (pStage) were determined according to the American Joint Committee on Cancer/International Union Against Cancer TNM staging system.

### 3.2 Clustering Analysis According to HERV and Genes Expression

Raw reads counted at cellular genes (*n* = 60 670) and HERV loci (*n* = 3280) were normalized and used to perform unsupervised clustering analyses, to evaluate samples distribution and grouping.

**Figure 1:**
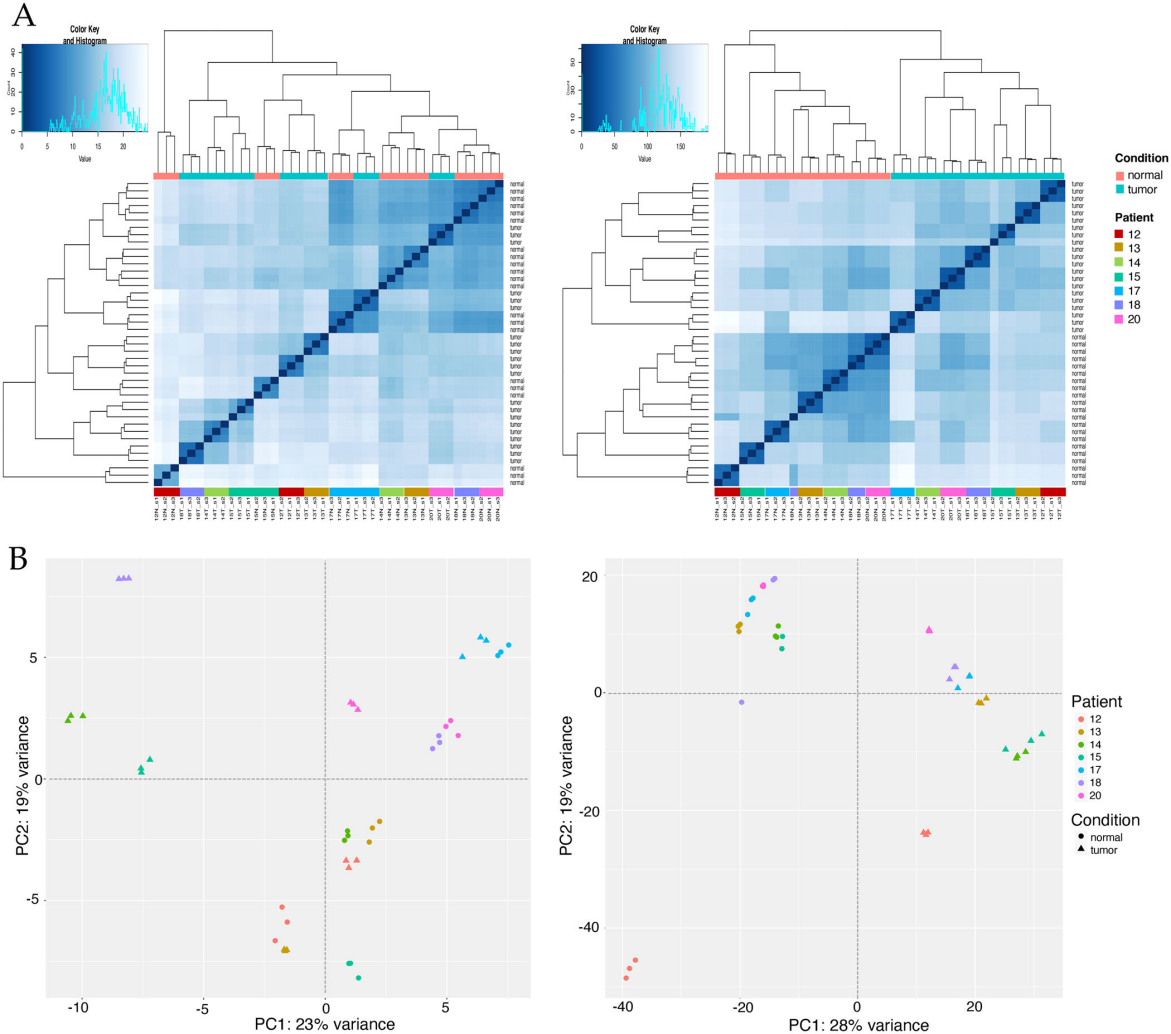
Unsupervised clustering analyses based on HERV and gene expression. *Panel A*. Heatmaps of pairwise distance among samples as calculated based on the overall HERV (left) and cellular gene (right) expression. *Panel B*. Principal Component Analysis (PCA). The first (PC1) and second (PC2) principal components of HERV (left) and cellular genes (right) transcriptional variance are shown at the zero of the x and y axis of each plot, respectively, along with the percentage of the overall variance they account for. In the genes plots, the PC1 is represented by the presence of the tumor, dividing tumor samples (right) from normal ones (left), while the PC2 corresponds to the gender, dividing patient 12 (male) from the others (all females).

Sample to sample distance plots based on HERV expression (Figure 1A, left) revealed the presence of a certain interindividual variability in the expression of HERVs in the different patients, since samples clustering was only partially based on the condition (tumor or normal tissue) and, in one case, both types of samples from the same individual clustered together (patient 17). Contrarily, when the same analysis was performed analyzing the cellular genes (Figure 1A, right) samples resulted to be divided in two clusters corresponding to normal and tumor tissues.

A principal component analysis (PCA) was performed to identify the first and second components responsible for HERV and genes transcriptional modulation among samples (PC1 and PC2, respectively) (Figure 1B). The analysis confirmed that the presence of CRC constitutes the major component of cellular genes' transcriptional modulation (28% of variance), since the PC1 clearly divides tumor from normal samples (Figure 1B, right). In addition, gender seems to account for the second component in genes' variance (PC2, 19%) with patient 12 (male) clustering apart from all the others (females). However, a higher number of male patients would be need to confirm such an impact of male gender on HERV expression. Concerning HERV PCA samples (Figure 1B, left), the presence of CRC seems not to be a principal component of HERV variance when considering all the patients together. At the light of the above interindividual variability in HERV expression, which could somehow mask the impact of the tumor condition, we decided to proceed with clustering analysis for the individual patients. In this case, the individual PCA confirmed that CRC is the principal component of HERV transcriptional modulation in all the patients, accounting for 58% to 94% of the overall variance in HERV expression (Figure S2).

**Figure 2:**
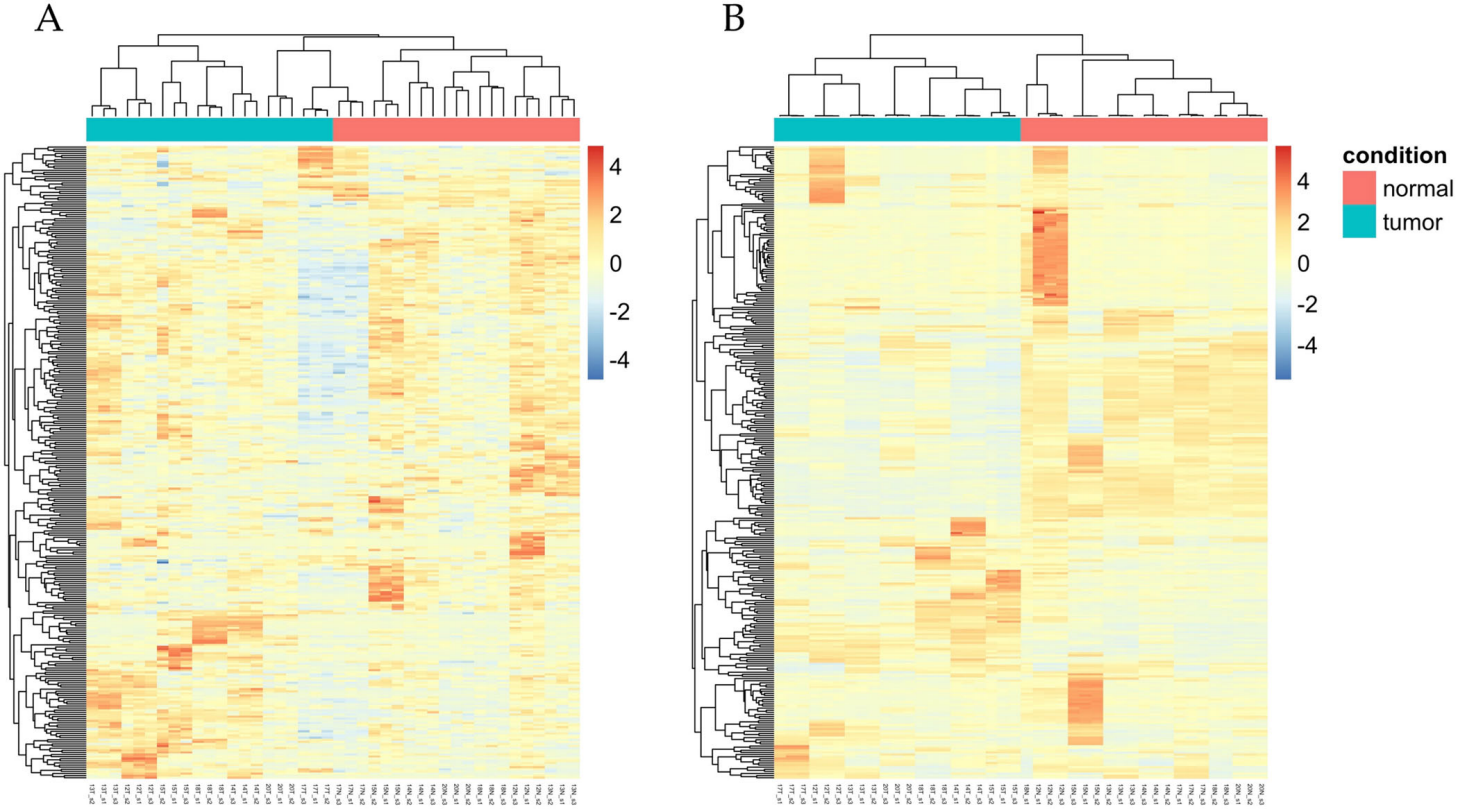
Heatmaps of the top 300 HERV and genes. The expression heatmaps were generated considering the top 300 HERVs (*panel A*) and cellular genes (*panel B*) as sorted by highest variance among samples. The dendrograms represent the clustering of patients (columns) and HERVs/genes (rows) according to such variance.

Accordingly, the generation of heatmaps based on the top 300 HERVs showing the highest variance among samples always grouped tumor samples separately from normal ones except for one individual (patient 17), whose normal samples clustered with the paired tumor ones (Figure 2A). Similarly, individual heatmaps based on the top 50 HERVs with the highest variance in each patient clearly divided tumor from normal samples (Figure S3). The analysis of the top 300 cellular genes confirmed the presence of two major clusters of patients, divided according to the CRC condition (Figure 2B).

### 3.3 Differential Expression (DE) Analyses

**Figure 3:**
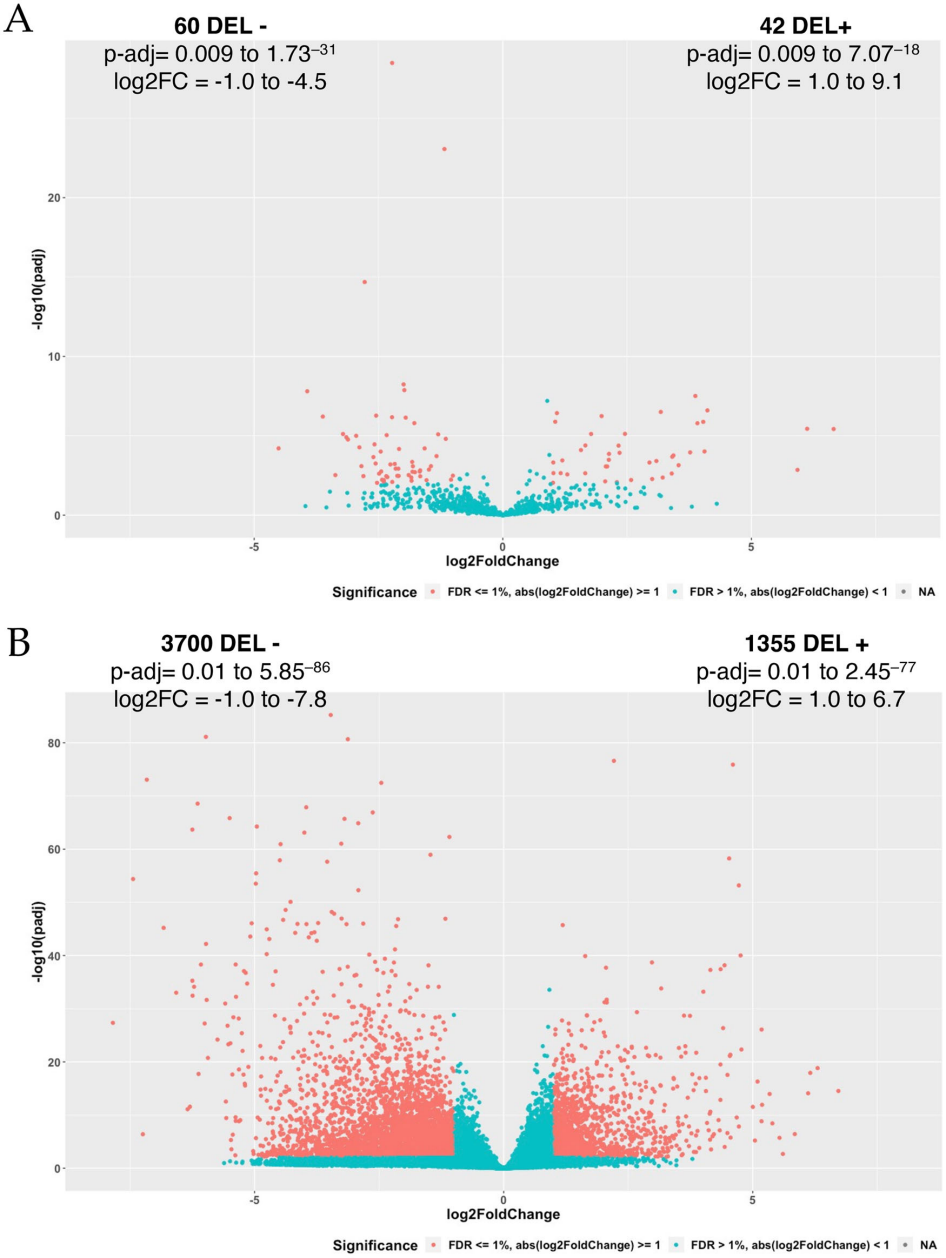
Volcano plots of HERVs and cellular genes. The plots represent the results of the differential expression analysis for HERV loci (*panel A*) and human genes (*panel B*): red dots correspond to the elements resulted significantly modulated in tumor samples as compared to normal tissues (FDR ≤ 1%, corresponding to an adjusted *p* ≤ 0.001); while blue dots indicates elements with a comparable expression among the two conditions. Among differentially expressed elements, upregulated (DEL+) and downregulated (DEL−) ones are further divided according to the positive and negative log2FoldChange, respectively.

**Figure 4:**
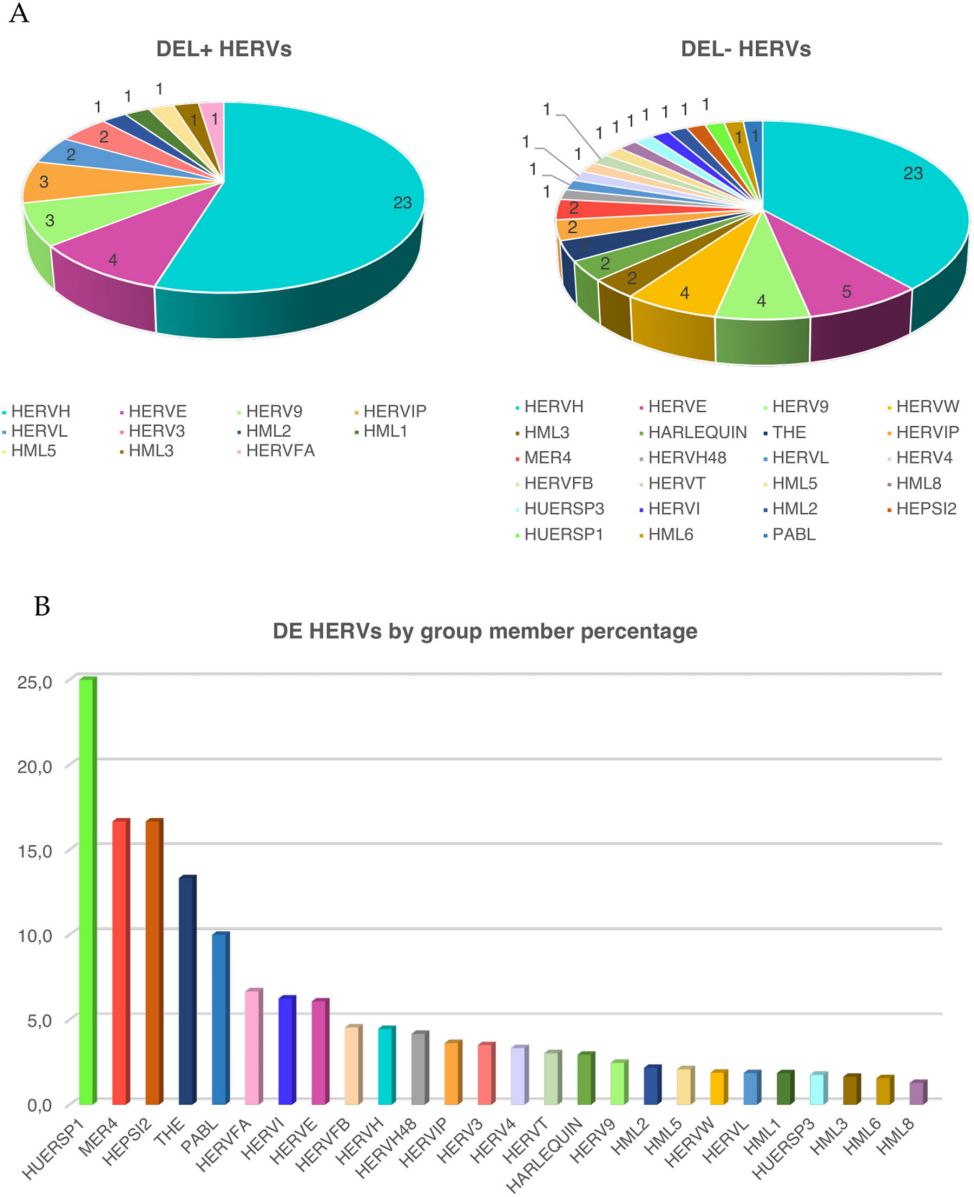
Overview of the deHERVs identified in CRC tissues. *Panel A*. Number of HERV loci found to be upregulated (left) or downregulated (right) in CRC condition and group of belonging. *Panel B*. Percentage of deHERVs for each group, as calculated based on the group total members took into account in the study.

A total DE analysis was performed with all the patients, evaluating HERVs and cellular genes modulation in tumor samples as compared with normal tissues. Despite the above mentioned basal interindividual variability, the analysis allowed to identify 102 HERVs (Figure 3A) and 5055 cellular genes (Figure 3B) modulated in CRC. Among them, 60 HERVs and 3700 genes were downregulated in the presence of the tumor, while the remaining 42 HERVs and 1355 genes were upregulated. 31 of the identified DE HERVs (30%, including 16 HERV‐H elements) were colocalized with 36 annotated genes that were protein coding in 14 instances (data not shown). The complete list of modulated HERVs and genes is provided as Table S1, and the 42 upregulated HERV elements are listed in Table 2. Interestingly, as shown in Figure 4, the 102 modulated HERVs belonged overall to 26 groups, with elements of the same group showing an opposite regulation. These included members of the highly investigated HERV‐H and HML2 groups (43 and 2 DE loci, respectively), already studied for a possible association with CRC, but also to other HERV groups never previously observed to be differentially expressed in CRC samples, such as HERV‐E (9 loci), HERV‐9 (8 loci), and HERV‐IP (5 loci) (Figure 4). Of note, a total of 23 specific HERV‐H loci were upregulated in CRC and represented hence the 55% of all upregulated HERV elements, in line with their abundance in the human genome.

**Table 2:** DE HERV loci upregulated in CRC tissues.

HERV	log2FC^1^	*p*adj^2^	Coordinates (hg38)	Group	Mean TPM^3^ (Normal)	Mean TPM^3^ (Tumor)
**5290**	**9.15**	**7.07E‐18**	**X:4540123‐4546198(−)**	**HERVH**	**0.06**	**46.03**
2003	3.87	3.11E‐08	5:139502943‐139512402(+)	HERV9	0.01	0.31
**4057**	**4.12**	**2.52E‐07**	**13:109265090‐109271115(+)**	**HERVH**	**0.08**	**2.00**
**4627**	**3.18**	**3.16E‐07**	**19:15828861‐15837304(+)**	**HERVH**	**0.17**	**2.12**
**4766**	**1.09**	**3.75E‐07**	**19:52649616‐52653116(+)**	**HERVIP**	**4.53**	**12.48**
1966	1.99	5.78E‐07	5:122475952‐122479132(−)	HERVH	0.18	1.03
**5346**	**1.05**	**1.31E‐06**	**X:37451928‐37461065(−)**	**HERVH**	**2.03**	**5.27**
1066	4.03	1.34E‐06	3:112690299‐112701577(+)	HERVL	0.02	0.34
4736	3.91	1.63E‐06	19:43321084‐43329393(−)	HERVH	0.05	0.99
**6196**	**6.12**	**3.67E‐06**	**1:221965965‐221971618(+)**	**HERVH**	**0.03**	**2.34**
**3987**	**6.65**	**3.76E‐06**	**13:66137809‐66147046(−)**	**HERVH**	**0.02**	**2.70**
5855	2.46	7.59E‐06	1:34630914‐34637213(−)	HERVH	0.05	0.39
**4849**	**1.78**	**7.70E‐06**	**20:49281128‐49287343(−)**	**HERVIP**	**0.71**	**3.10**
546	1.66	4.07E‐05	2:58113432‐58119130(−)	HERVH	0.25	0.93
3527	2.33	4.20E‐05	11:67845549‐67851894(−)	HERVE	0.03	0.20
3542	1.57	7.97E‐05	11:71740712‐71749874(+)	HERVE	0.07	0.24
4098	4.06	9.77E‐05	14:31240557‐31251389(−)	HERVH	0.03	0.68
5305	3.77	0.00011	X:16175752‐16184467(−)	HERVH	0.03	0.41
5361	2.35	0.00012	X:49139779‐49148918(−)	HERVE	0.09	0.48
6069	2.14	0.00014	1:155626674‐155635703(−)	HML2	0.05	0.22
4905	3.43	0.00017	21:42800846‐42806578(−)	HERVH	0.04	0.52
914	3.41	0.00021	3:34518837‐34524418(−)	HERV9	0.02	0.32
3066	2.13	0.00033	9:64870613‐64878934(−)	HERVIP	0.08	0.44
4744	1.20	0.00036	19:47047398‐47055397(−)	HERVH	0.85	1.81
4270	3.09	0.00039	15:51282241‐51286694(+)	HML1	0.01	0.22
**2521**	**1.02**	**0.00048**	**7:64990455‐64999936(−)**	**HERV3**	**3.06**	**7.74**
2565	2.95	0.00049	7:93639935‐93645553(+)	HERVH	0.05	0.36
3733	3.54	0.00072	12:20937062‐20947112(−)	HERVE	0.00	0.10
6205	2.07	0.00085	1:225221769‐225229271(+)	HML5	0.02	0.12
2080	2.10	0.00086	6:16259015‐16264895(−)	HERVH	0.06	0.33
**1999**	**5.93**	**0.00141**	**5:136540941‐136543623(−)**	**HERVH**	**0.04**	**3.20**
**2476**	**1.02**	**0.00226**	**7:43853008‐43866752(−)**	**HML3**	**3.93**	**8.47**
3113	1.16	0.00227	9:94539871‐94545510(+)	HERVH	0.30	0.82
4050	1.66	0.00230	13:99488624‐99496752(−)	HERV9	0.08	0.28
2897	3.40	0.00240	8:90089333‐90095881(−)	HERVH	0.03	0.39
5480	1.30	0.00272	X:74121599‐74130555(−)	HERVH	0.43	1.31
4825	3.22	0.00429	20:19752022‐19756780(−)	HERVH	0.15	1.52
3165	3.00	0.00535	9:129587224‐129596563(−)	HERVFA	0.01	0.14
5705	2.58	0.00614	X:131712222‐131717768(+)	HERVH	0.04	0.24
2895	2.06	0.00747	8:89448803‐89455243(+)	HERVL	0.03	0.15
2955	2.29	0.00837	8:127431701‐127440906(+)	HERVH	0.03	0.18
2648	1.01	0.00917	7:138556758‐138566149(−)	HERV3	0.10	0.24

*Note:* The DE HERV loci selected for transcriptome de novo reconstruction are highlighted in bold.

^1^ Log2 Fold Change with respect to normal tissue.

^2^ Benjamini‐Hochberg adjusted *p* value (threshold: padj < 0.001).

^3^ Transcripts per Million Kilobases.

**Figure 5:**
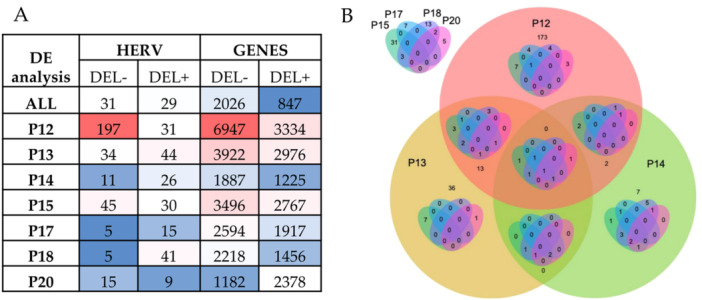
Results of patient‐specific DE analyses ***.** Panel A*. Results of the DE analysis for HERV and genes as conducted for the whole data set (ALL) as well as for the individual patients, comparing for everyone the three tumor samples to the three normal tissue samples. The table cells are colored according to the number of modulated HERV and genes (increasing from blue to red). *Panel B*. Nest Venn diagram representing the modulated HERVs shared by the different individuals.

To evaluate the level of interindividual variability in HERV modulation, and to identify the subset of DE HERV that are commonly modulated in all the individuals, the above results have been compared with the ones obtained performing the DE analysis in each individual between the respective tumor and normal tissue samples (Figure 5). As suggested from clustering analyses, the different patients were interested by a certain variability in genes and HERV expression: the individual with the highest number of modulated elements was patient 12, that is, the only male, with a total of 228 HERVs and 10281 genes modulated in proximal sigmoid CRC (Figure 5A). Conversely, the patients with the lowest number of modulated HERV and genes were respectively patient 17 (ascending colon, 20 deHERVs) and patient 14 (rectum, 3112 DE genes). A single HERV locus was found to be modulated in all individual analyses (Figure 5B). The HML3 element on chromosome 17q21.32 (HERV ID = 4463) was in fact downregulated in all CRC tissues as compared with the respective control samples.

### 3.4 Transcriptome Reconstruction and qRT‐PCR Validation of HERVH‐5290

To evaluate the putative transcript produced by the identified DE HERVs, we performed a de novo transcriptome reconstruction for 11 elements found to be upregulated in CRC samples and showing a TPM ≥ 2 in the tumor samples (highlighted in bold in Table 2).

**Figure 6:**
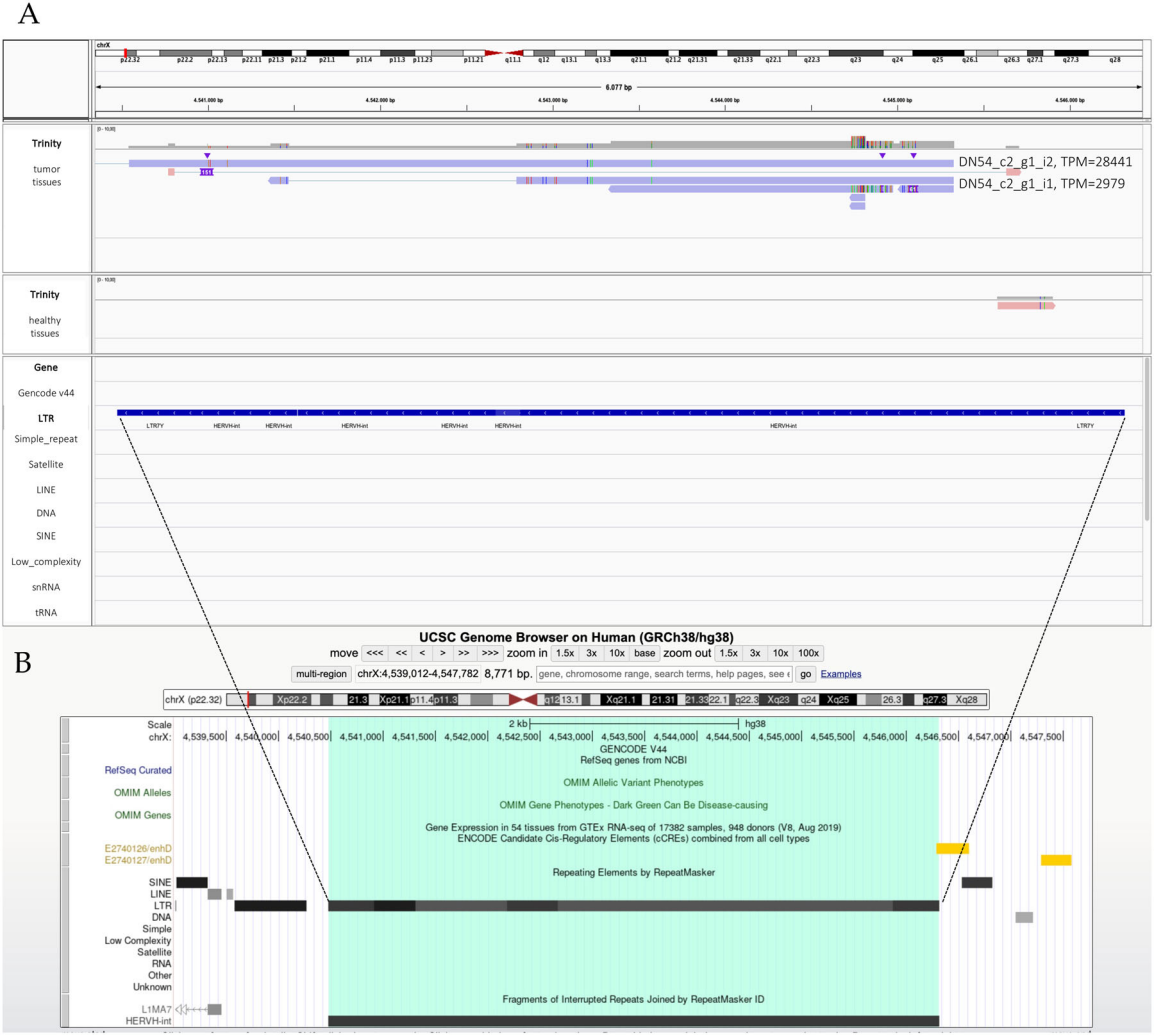
Transcript reconstruction for the HERVH locus 5290. *Panel A*. The transcripts inferred for the HERVH locus showing the highest TPM in CRC tissues are visualized in the Integrative Genome Browser (IGV) for tumor (up) and normal (down) conditions, along with the annotations for cellular genes (Gencode v44) and repetitive elements (RepeatMasker) in the lowest part of the panel. *Panel B*. The structure of the HERVH locus in the context of Genome Browser.

Among the reconstructed transcripts (Figure S4), the majority were found to be specific for the tumor, being absent or very fragmented in normal tissue. Particularly, the transcripts inferred for the HERV‐H on Xp22.33 chromosome (HERV ID 5290, first by TPM value in CRC tissues, Table 2) had a complete structure and showed a high expression in tumor samples, being not found in the healthy tissue counterpart (Figure 6). This is in line with the strong upregulation of the locus in CRC, with an adjusted *p* value of 7.07^−^^18^ and a log2 fold change of 9.15 (Table 2), highly significant also given the fact that it is devoid of colocalized cellular genes that could influence its expression. Of note, this transcript DN54_c2_g1_i2 was uniquely found in CRC samples (TPM 28441). It is 5538 bp in length and spans the entire HERV‐H locus, including a predicted ORF potentially coding for a Pol‐derived peptide of 173 aa (Figure 6). Importantly, the same HERV‐H element predicted to produce this transcript has been repeatedly described to be upregulated in CRC and suggested as ﻿a possible target of immunological interventions [12, 34]. To validate its upregulation in tumor tissues, a specific primer pair was designed on the identified transcript and used to test its expression by quantitative real‐time PCR in five pairs of the sequenced specimens, confirming its significant increase in CRC (*p* ≤ 0.01, Figure S5).

## 4 Discussion

﻿CRC is a multifactorial disease with poorly understood etiology, representing a leading cause of death worldwide. As for other tumors, the search for genetic hallmarks and therapeutic targets also includes the analysis of HERV expression since these elements are often reported as upregulated in tumor tissues and have the potential to induce and sustain cellular transformation. Despite this, the oncogenic activity of HERVs is still poorly demonstrated, and most of the studies investigated the expression of whole HERV groups with approaches that often are not able to sufficiently distinguish the specific contribution of the individual members due to their high nucleotide identity (e.g.,: PCR, probes) [35]. In this regard, high‐throughput approaches as RNA‐seq may provide a reliable information about the individual HERV loci activated in tumor tissues, being based on the univocal genomic position of each element in the human genome [36]. In the present study, we applied RNA‐seq analysis to the characterization of the HERV transcriptome in a population of 7 CRC patients to define a more complete and detailed picture of the HERV expression patterns, identifying the HERV loci modulated in the tumor. Of course, two limits of the present study regards the small sample size ‐ also in light of the existing interindividual variability ‐ and the low representation of the male gender in our population (1 individual). For this reason, the above high througput approach was chosen, and sequencing parameters were optimized to assure the highest number of reads being univocally mapped to highly similar HERV elements.

Our results, including the analysis of the top 300 HERV by variance, confirm that CRC is a major factor in driving gene transcriptional variance. This was somehow expected, since the presence of the tumor is well known to have a major impact on cellular transcription. Interestingly, HERV expression analysis confirmed that there is a relevant basal interindividual variability, which is also in line with the reported genetic intratumoral and intertumoral heterogeneity resulting from the accumulation of genetic mutations and chromosomal aberrations during disease initiation and progression [34]. Performing individual analyses, to compare tumor and normal tissues from the same CRC patients, further confirms that the tumor condition has a major impact on HERV expression, and is likely influenced by the individual stage and localization.

Accordingly, the analysis of DE genes and HERV as performed for each patient confirms a certain interindividual variability in their modulation in CRC tissues. For both sets, the highest modulation was observed in CRC tissue from patient 12, possibly suggesting that a proportion of such modulation could be associated to male gender. However, this could also depend on the tumor site, since the second in terms of HERV and genes modulation was patient 13 (78 and 6898, respectively), who also had a low degree sigmoid tumor. In this regards, the study by Golkaram et al. reported that HERVs show specific molecular subtype in a cohort of 114 patients with stage II and III CRC, observing a correlation between the elevated expression of HERVs and the low responsivity to chemotherapy, with a subsequent poor clinical outcome [14]. Even if assessing the role of these HERV in CRC was out of the scope of the paper, they proposed that HERV median expression can be exploited as potential biomarker for prognosis, relapse, and unfavorable chemotherapy responses median expression of hERVs can serve as a potential biomarker, and resistance to chemotherapy in stage II and III CRC [14]. Specific comparison with the results of the present study is hard to make due some relevant differences in Golkaram et al. results experimental design, since in their case (i) whole transcritpome sequencing was performed for tumor samples only, with paired end reads that were significantly shorter as compared to the present study (76 bp) and (ii) HERV expression in tumor samples was reported as counts per million (CPM), which are normalized according to sequencing depth only, without considering each HERV length as for TPM.

Of note, in half of the studied individuals (4/8), the Syncytin locus (HERV‐W 7q21.1), whose Env product has the potential to contribute to transformation and invasion processes through its fusogenicity [3, 37, 38], is expressed. In these samples, however, HERV‐W 7q21.1 shows a basal transcription that is actually reduced in tumor samples as compared to normal ones (mean TPM of 0.8 ± 0.6 and 1.4 ± 0.9, respectively). Syncytin immunoreactivity has been previously observed at different degrees in CRC patients, being associated with decreased overall survival in rectal but not in colonic cancer patients [39]. Even if present results do not demonstrate a relevance for Syncytin expression in CRC condition, it is worth to note that the highest TPM values in tumor tissues were observed for patients 13 and 14, having a sigmoid and rectal localization. Further studies on these tumors should be implemented to better define Syncytin's potential role in CRC.

In any case, despite the above mentioned basal interindividual variability, the differential expression analysis allowed to identify 102 HERVs and 5055 cellular genes modulated in CRC, with a prevalence of downregulation in both cases (59% and 73%, respectively). Of note, the modulated HERVs not only belongs to the groups already investigated for CRC, namely HERV‐H and HML2, but also to additional 24 clades not previously investigated in this context and observed to be modulated in the present study for the first time, particularly HERV‐E, HERV‐9, and HERV‐IP. Indeed, among the 42 upregulated HERVs, 23 are HERV‐H loci, confirming the relevance in CRC of some members of this numerous group, with 7 of them showing high TPM in CRC samples.

As already stated, HERV‐H loci expression has been reported throughout CRC progression, especially for evolutionarily‐young HERV‐H subfamilies whose 5'LTR often presented active histone marks [12]. In previous reports, microarray analysis in ﻿4 pairs of tumor and adjacent normal tissues [40] led to the identification of the 21 HERV‐H loci that are differentially expressed in tumors and their transcriptional activity has been studied by RT‐PCR leading to a short list of five HERV‐H sequences with potential relevance for CRC. In a subsequent study, the latter ﻿showed a variable pattern of expression when evaluated in 139 CRC samples, 100 of which were positive for the expression of one to all five of them, further emphasizing the transcriptional heterogeneity of HERV‐H members [12]. In this scenario, present results demonstrate that, contrarily to the general idea that the whole HERV‐H group is upregulated in the CRC context, the same number of HERV‐H loci (23) is downregulated or upregulated in the tumor tissue, indicating that there is a specific modulation at the locus level and not a general modulation of the whole group. Such results also point out to the fact that single HERV expression is likely influenced by its genetic context of integration and by the eventual epigenetic alterations induced by the tumor. In line with this, a recent study reported an ﻿increased expression of HERV‐H full‐length elements in tumor tissues from the TCGA colorectal data set (COREAD), which was correlated with the mutation of several genes [13]. ﻿siRNA knockdown of some of these genes resulted in increased transcription of the HERV‐H locus, including ﻿ARID1A, ﻿a core subunit of BAF chromatin remodeler complex whose mutational inactivation is broadly found in human cancers [13]. The loss of this tumor suppressor was reported to induce the transcriptional de‐repression of at least 25 HERV‐H loci, and the resulting ﻿HERV‐H transcripts colocalize with nuclear BRD4 foci and influence the downstream transcriptional network [13]. Comparing these results with our study, we confirm that the HERV‐H in locus Xp22.2 is upregulated, showing however a weak activation according to TPM values, leaving open the question of its relevance. More interestingly, among the 23 upregulated HERV‐H elements reported in the present study, 16 were colocalized with annotated genes and 6 of these code for proteins that include TMEM160 and VKR‐2. TMEM160 inhibits the degradation of PD‐L1 that, in turn, fosters the malignant progress, radio‐resistance and immune evasion of CRC cells [41], while VKR‐2 is a kinase effector of signaling pathways that regulate apoptosis and tumor cell growth, also through PD‐L1 network [42]. Hence, we show that the activation of these HERV‐H loci is linked to the modulation of such cascade in CRC samples. Further studies are needed to better understand the role of the HERV‐H sequences in the modulation of the TMEM160 and VKR‐2 expression.

With regard to the other HERV group previously investigated in CRC, namely HERV‐K(HML2), according to our results only 2 elements were differentially expressed in CRC samples, showing opposite modulation. A recent study reported ﻿that ﻿the transcript levels of HERV‐K(HML‐2) *gag*, *pol*, and *env* genes were significantly upregulated in CRC [43], but further comparison with the present study is prevented by the fact that authors used probes and primers with unknown range of specificity among the around 90 HML2 elements present in the human genome [43]. A second study investigated the role of the group by ﻿obtaining a knockout of an HERV‐K(HML2) *env* gene (K119, at chr12:58 721 197–58 722 612) in CRC cell lines, observing a decrease in cellular proliferation and invasion in in vitro and in nude mice models [44]. These effects were linked to the ﻿significant downregulation of nuclear protein‐1 ﻿(NUPR1) in HERV‐K(HML2) knockout cells, suggesting that the HERV‐K *env* gene alters tumorigenic characteristics through this ﻿transcriptional regulator pathway [44]. However, neither this HML2 locus nor NUPR1 was modulated in our samples, possibly suggesting a more heterogenous and complex situation in vivo.

To evaluate the actual transcript production by the identified upregulated DE HERVs, a de novo transcriptome reconstruction was performed for 11 elements showing a TPM ≥ 2 in CRC samples, among which the one showing the highest upregulation in the tumor was a HERV‐H locus on Xp22.3 (HERVH‐5290, TPM = 46 in CRC vs 0 in healthy speciments). ﻿Previous data described by qRT‐PCR that this HERV‐H locus on Xp22.3 is upregulated in CRC and other human tumors [12, 34], retaining residual promoter activity in its 5’ U3 region [45]. Interestingly, the transcripts we inferred for HERVH‐5290 locus shows a high level, specific expression in tumor samples. In particular, transcript DN54_c2_g1_i2 is uniquely found in CRC samples and shows a TPM of 28441. The same locus has been associated to the expression of a derived *gag* transcript in cancer lesions, possibly encoding a truncated protein of 93 amino acids that has been proposed as ﻿a possible target of immunological antitumor therapy [34]. In our case, the inferred transcript was 5538 bp in length and spanned across *gag*, *pol*, and *env* regions, including a predicted ORF that encoded for a Pol‐derived peptide of 173 aa. After the RNA‐seq was performed, we were able to validate HERVH‐5290 upregulation by qRT‐PCR on five pairs of CRC samples that had enough amount of remaining RNA, and we confirmed a significant upregulation of this locus. Dedicated studies should be performed to assess whether the derived peptide is produced in CRC tissues and to characterize its molecular effects on tumor cells and gene expression, to further evaluate the potential of this HERV‐H locus as buomarker and innovative therapeutic target for CRC.

## Author Contributions

Enzo Tramontano and Liang‐Tzung Lin conceived and supervised the study, Nicole Grandi performed the bioinfomatic analyses and wrote the manuscript, Ching‐Hsuan Liu took care of sample preparation and wet lab experiments and participated in the writing, Yun Yen curated the cinical sample collection and related ethical aspects, Saili Chabukswar and Daniele Carta participated to data analysis. All authors contributed to the final version of the manuscript.

## Ethics Statement

The study was conducted in accordance with the Declaration of Helsinki, and approved by the Joint Institutional Review Board of Taipei Medical University.

## Consent

Informed consent was obtained from all subjects involved in the study.

## Conflicts of Interest

The authors declare no conflicts of interest.

## Supporting information


**Figure S1. Quality of the RNA‐seq profiles generated in the study**. Quality check has been performed with FastQC (version 0.11.8) on raw read paired fastq files for each sample. Results have been aggregated using MultiQC modular tool (version 1.7).

**Figure S2. Principal Component Analysis (PCA) of HERV expression variance**. The first (PC1) and second (PC2) principal components of HERV transcriptional variance are shown at the zero of the *x* and *y* axis of each plot, respectively, along with the percentage of the overall variance they account for. In all the plots, the PC1 is represented by the presence of the tumour, dividing tumour samples (right) from normal ones (left).

**Figure S3. Heatmaps of the top 50 HERVs as sorted by variance among samples**. The dendrograms represent the clustering of patients (columns) and HERVs (rows) according to such variance.

**Figure S4. Transcripts reconstructed for the deHERVs others than HERVH‐5290 showing high TPM in CRC samples**. in each panel, the transcripts inferred with Trinity for the selected deHERVs in tumour (top) and normal (bottom) tissues are shown in the context of the human genome. Annotations for cellular genes (Gencode, version 44) and LTR‐retrotransposons (RepeatMasker) are shown in blue in the lowest part of the panel. The deHERV with the highest TPM (HERVH 5290), is shown in Figure 6.

**Figure S5. qPCR validation of HERVH‐5290 RNA levels in CRC samples**. Quantitative real‐time PCR was performed in five pairs of the sequenced CRC specimens using the following primers: forward 5′‐TCCTCAATACCTCCCTCTACTACC‐3′, reverse 5′‐GGGATGAAGGGTGCAAAGGA‐3′. Statistical significance (**p ≤ 0.01) was determined by Mann–Whitney test using GraphPad Prism 9.

Supporting information.

## Data Availability

The RNA‐seq profiles generated in the present study are available on request.
