# Supplementary figures and images for: HERV Modulation in Colorectal Carcinoma Patients: A Snapshot of Endogenous Retroviral Transcriptome
Source: J Med Virol. 2025 Feb 24;97(3):e70249. doi: 10.1002/jmv.70249 (PMC11849272; doi:10.1002/jmv.70249)

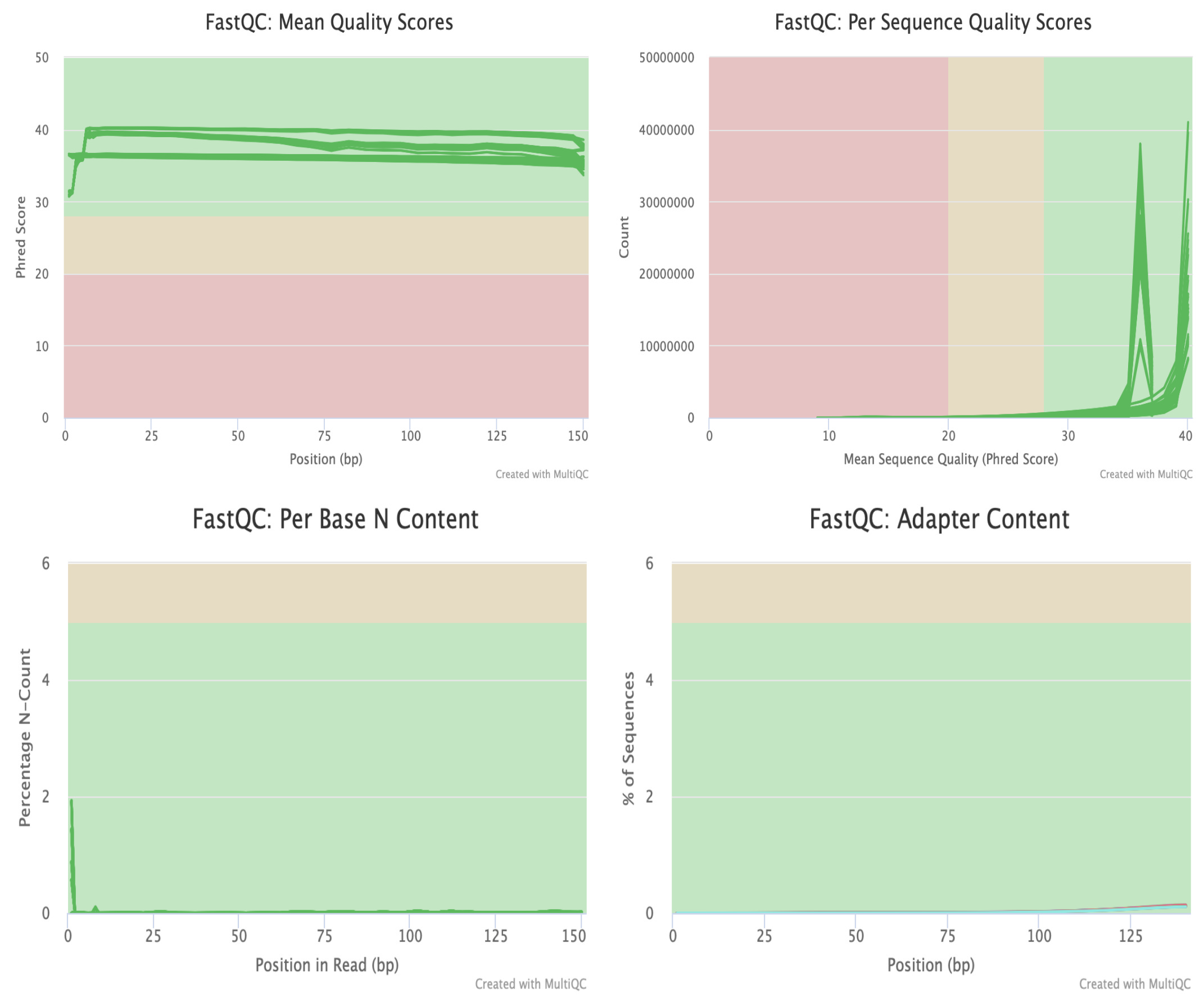

Supplement: Supplementary file 1 — Figure S1. Quality of the RNA‐seq profiles generated in the study. Quality check has been performed with FastQC (version 0.11.8) on raw read paired fastq files for each sample. Results have been aggregated using MultiQC modular tool (version 1.7). [file JMV-97-e70249-s003.tif]

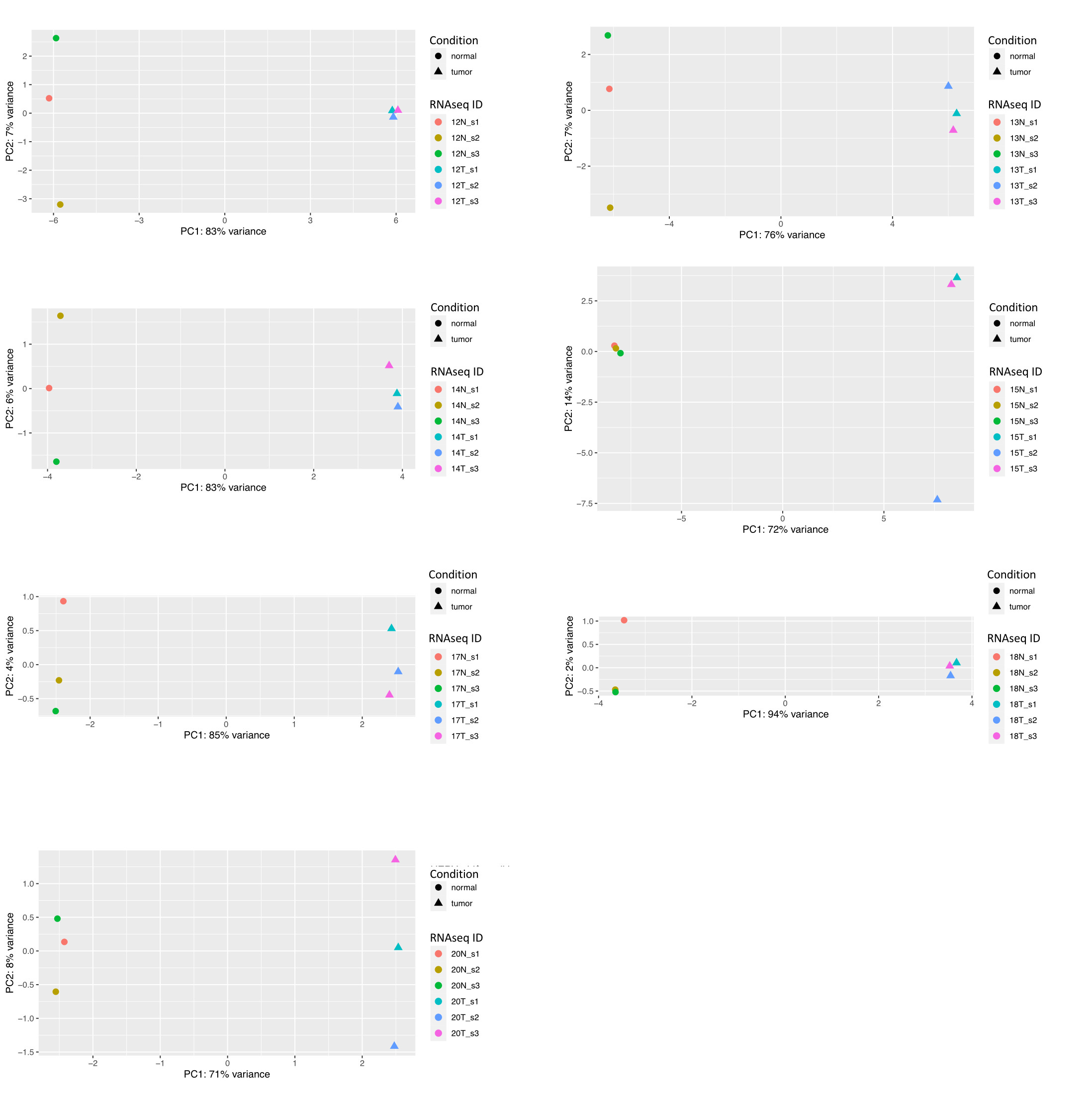

Supplement: Supplementary file 2 — Figure S2. Principal Component Analysis (PCA) of HERV expression variance. The first (PC1) and second (PC2) principal components of HERV transcriptional variance are shown at the zero of the x and y axis of each plot, respectively, along with the percentage of the overall variance they account for. In all the plots, the PC1 is represented by the presence of the tumour, dividing tumour samples (right) from normal ones (left). [file JMV-97-e70249-s006.tif]

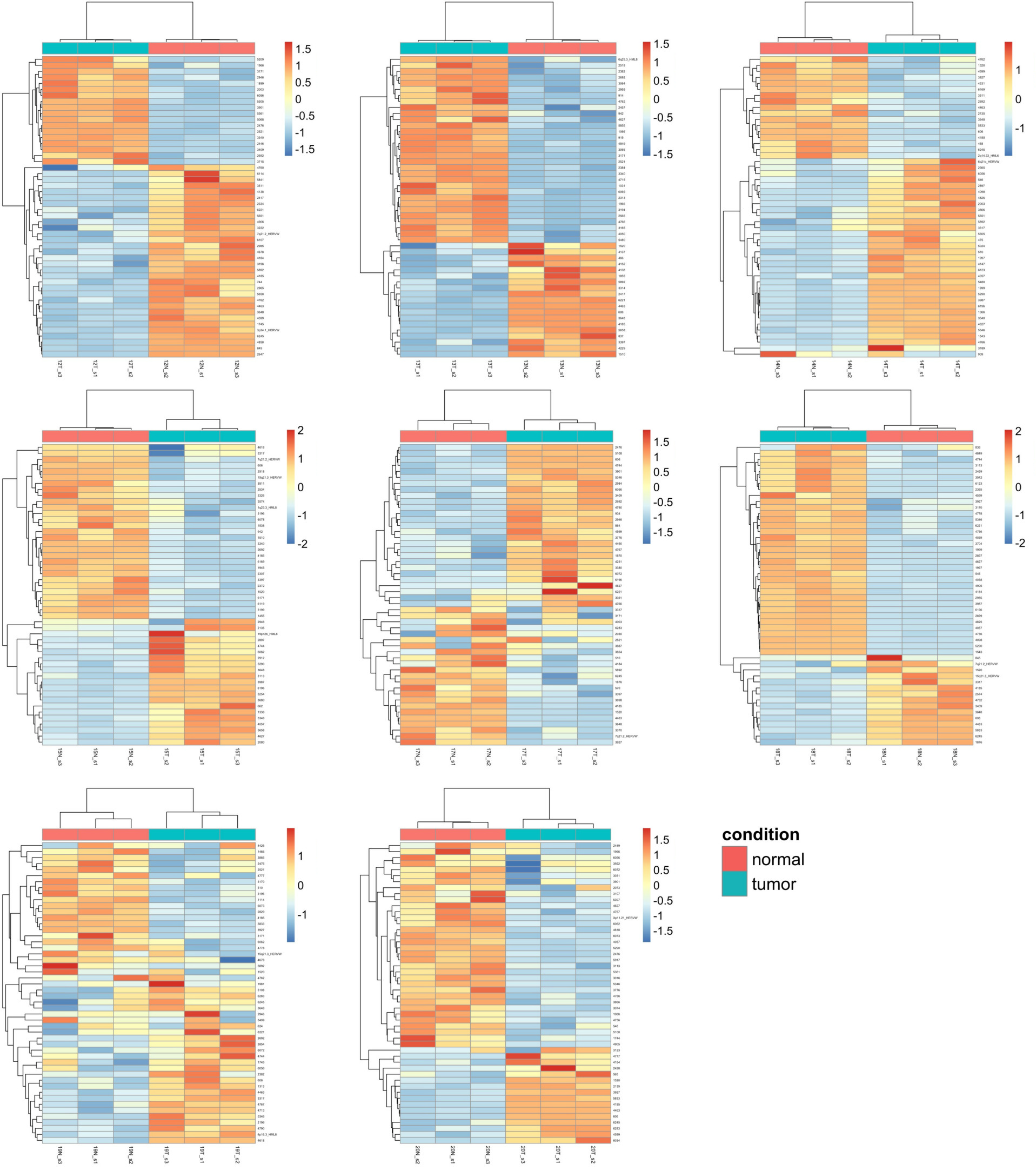

Supplement: Supplementary file 3 — Figure S3. Heatmaps of the top 50 HERVs as sorted by variance among samples. The dendrograms represent the clustering of patients (columns) and HERVs (rows) according to such variance. [file JMV-97-e70249-s005.tif]

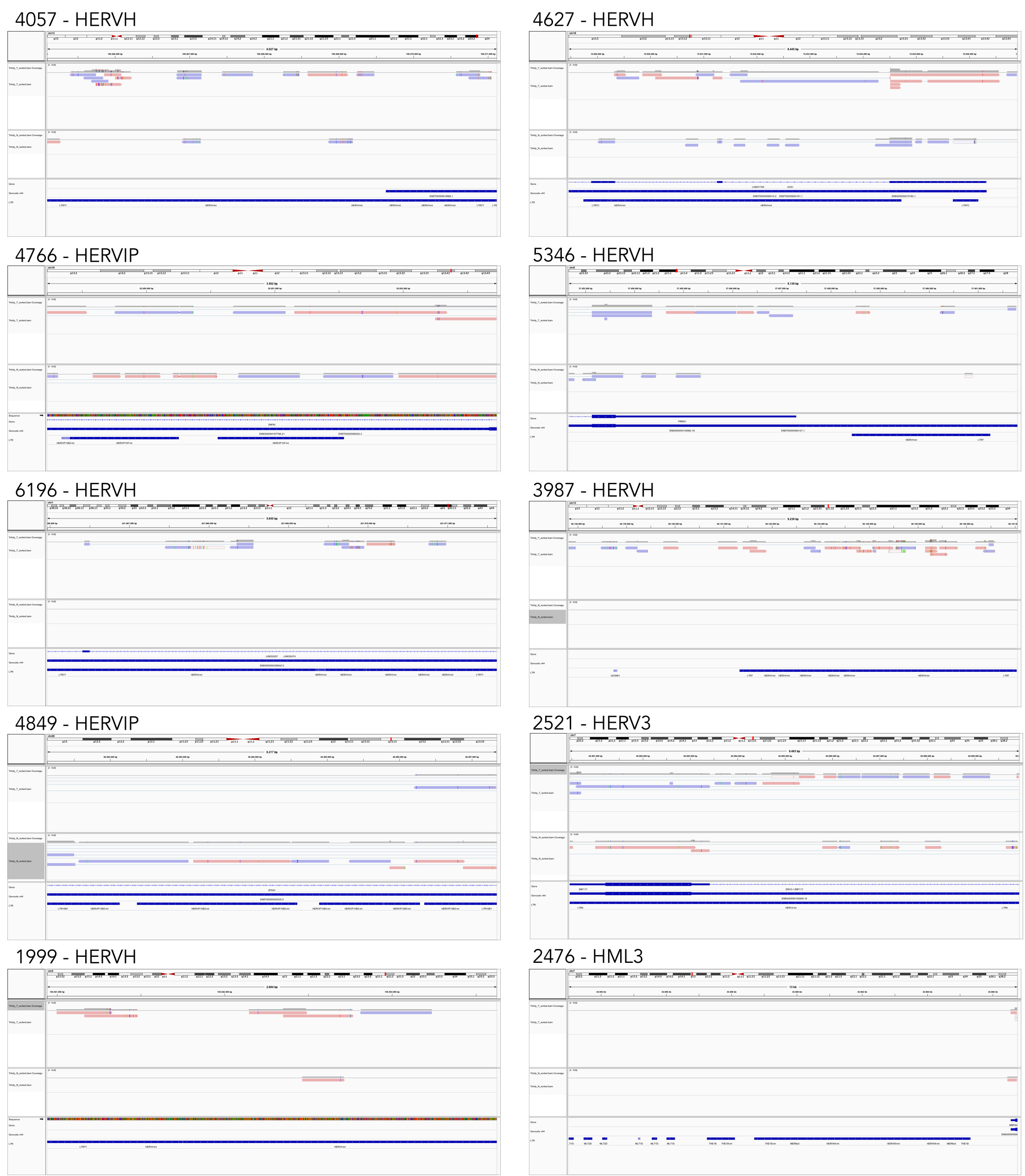

Supplement: Supplementary file 4 — Figure S4. Transcripts reconstructed for the deHERVs others than HERVH‐5290 showing high TPM in CRC samples. in each panel, the transcripts inferred with Trinity for the selected deHERVs in tumour (top) and normal (bottom) tissues are shown in the context of the human genome. Annotations for cellular genes (Gencode, version 44) and LTR‐retrotransposons (RepeatMasker) are shown in blue in the lowest part of the panel. The deHERV with the highest TPM (HERVH 5290), is shown in Figure 6. [file JMV-97-e70249-s001.tif]

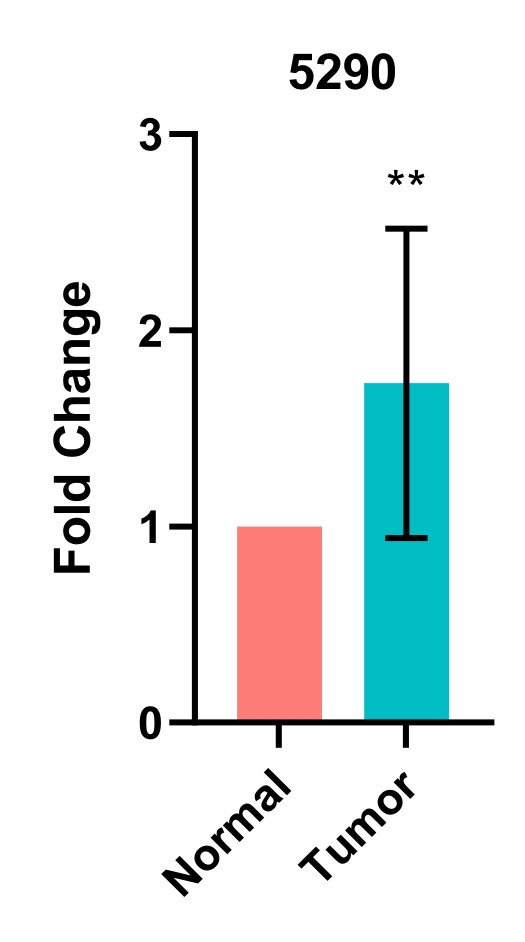

Supplement: Supplementary file 5 — Figure S5. qPCR validation of HERVH‐5290 RNA levels in CRC samples. Quantitative real‐time PCR was performed in five pairs of the sequenced CRC specimens using the following primers: forward 5′‐TCCTCAATACCTCCCTCTACTACC‐3′, reverse 5′‐GGGATGAAGGGTGCAAAGGA‐3′. Statistical significance (**p ≤ 0.01) was determined by Mann–Whitney test using GraphPad Prism 9. [file JMV-97-e70249-s004.tif]
